# Short-term outcomes of intravitreal faricimab on refractory neovascular age-related macular degeneration patients in China

**DOI:** 10.3389/fmed.2026.1855248

**Published:** 2026-06-19

**Authors:** Fen Zhou, Wen Bai, Yue Zhao, Qin Jiang, Chenghu Wang, Xiangzhong Xu, Jin Yao

**Affiliations:** Nanjing Medical University Affiliated Eye Hospital, Nanjing, China

**Keywords:** Ang-2, anti-VEGF, faricimab, IRF, nAMD, SRF

## Abstract

**Purpose:**

To assess the real-world clinical outcomes of faricimab in patients receiving treatment for neovascular age-related macular degeneration (nAMD).

**Methods:**

This real-world prospective observational study included patients with refractory nAMD. In total, 37 patients were analyzed. All patients had previously undergone at least three consecutive intravitreal injections of other anti-vascular endothelial growth factor (anti-VEGF) agents. The primary outcomes were best-corrected visual acuity (BCVA) and anatomical outcomes on optical coherence tomography angiography (OCTA) at a four-month follow-up period.

**Results:**

The faricimab regimen resulted in significant improvements in both visual and anatomical parameters among patients with refractory nAMD. LogMAR BCVA decreased substantially from 0.82 ± 0.42 to 0.57 ± 0.32 (*p* < 0.001). Central subfield thickness (CST) was reduced significantly from 336.89 ± 141.52 μm to 244.19 ± 69.91 μm (*p* < 0.001). The pigment epithelial detachment (PED) area decreased from 1.30 ± 0.71 to 0.94 ± 0.55 mm^2^ (*p* < 0.001), and the choroidal neovascularization (CNV) area shrank from 1.11 ± 0.64 to 0.97 ± 0.56 mm^2^ (*p* < 0.001). Both the subretinal fluid (SRF) and SRF & Intraretinal fluid (IRF) subgroups exhibited notable improvements in both vision and anatomical structure. Eyes with isolated SRF achieved longer dosing interval extensions (66.7% reached ≥3 months) compared to the SRF & IRF group (47% reached ≥3 months).

**Conclusion:**

Faricimab given as three monthly loading doses demonstrates significant short-term improvement in vision and anatomical outcomes, including rapid gains in BCVA and reductions in CST and retinal fluid. It appears effective and well-tolerated for refractory nAMD, particularly in reducing SRF and improving visual acuity, though its effect on IRF resolution was limited and not statistically significant. However, because the study design confounded drug switch with increased injection frequency, the independent contribution of faricimab itself cannot be determined. No adverse safety signals were observed.

## Introduction

Age-related macular degeneration (AMD) is a principal cause of irreversible blindness globally (1, 2). Neovascular age-related macular degeneration (nAMD) represents the advanced stage of disease progression, leading to severe vision impairment (3). The overall burden of nAMD is expected to grow, with prevalence projected to rise from 196 million in 2020 to 288 million in 2040 (4). Its growing public health impact has even drawn attention on digital health education platforms such as TikTok (5). Despite increased disease awareness, the management of nAMD, particularly in refractory cases, continues to pose a significant clinical challenge. Although anti-vascular endothelial growth factor (anti-VEGF) therapy has substantially advanced nAMD management, about 30% of patients exhibit suboptimal responses, characterized by persistent subretinal fluid (SRF) or intraretinal fluid (IRF) (6, 7). This refractory nAMD subgroup is at elevated risk of progressive visual loss, heightened treatment burden, and impaired quality of life. Therefore, optimizing therapeutic strategies for these patients is crucial. Beyond vascular endothelial growth factor (VEGF)-driven angiogenesis, angiopoietin-2 (Ang-2) has emerged as another key mediator in nAMD pathogenesis, contributing to vascular instability, leakage, and inflammation. Targeting both pathways simultaneously may offer a novel approach for refractory nAMD (8, 9).

Faricimab is the first bispecific antibody to simultaneously inhibit both Ang-2 and VEGF pathways (10, 11). Ang-2 is integral to regulation of vascular stability, angiogenesis, and vascular permeability, and its interplay with VEGF-A amplifies abnormal neovascularization and leakage in nAMD (12). Landmark phase 3 trials (TENAYA and LUCERNE) established the efficacy of faricimab in treatment-naïve nAMD, with a higher proportion of patients achieving freedom from retinal fluid (RF) (10). The TRUCKEE study further substantiated faricimab’s effectiveness in both treatment-naïve and previously treated nAMD populations (13). Pandit et al. demonstrated that intravitreal faricimab may not only improve anatomic outcomes in patients with previously treated nAMD, but also maintain stable vision in the short-term. Notably, reductions in fibrovascular pigment epithelial detachment (PED) height, central foveal thickness, SRF, and IRF were observed (14). Although the dual inhibition of Ang-2 and VEGF represents a promising therapeutic strategy, real-world evidence specifically in Chinese patients with refractory nAMD remains limited. To date, no study has reported on faricimab treatment outcomes in this specific population.

This study aims to investigate early outcomes in patients with refractory nAMD undergoing a switch to faricimab therapy, evaluating its efficacy and potential as a longer-lasting therapeutic alternative.

## Materials and methods

### Study participants

Written informed consent was obtained from all participating patients prior to enrollment. This real-world prospective observational study was approved by the Ethics Committee of the Eye Hospital of Nanjing Medical University (approval No. 2025009) and enrolled patients with refractory nAMD who were switched to faricimab at the same institution between July 2024 and June 2025. For patients with unilateral nAMD, the affected eye was included. For patients with bilateral active nAMD, the eye with worse baseline BCVA was selected as the study eye to ensure that the more severely affected eye received protocol-directed treatment and assessment. Refractory nAMD was defined as persistent IRF or SRF or enlarging PED despite ≥3 consecutive prior anti-VEGF injections and inability to extend injection interval beyond 6 weeks. Recalcitrance criteria included enlarging PED, IRF or SRF during attempts to extend the interval, or persistent SRF/IRF despite a four-week injection schedule. Inclusion criteria comprised: (1) age 55–90 years; (2) fluorescein angiography confirming active choroidal neovascularization (CNV) encompassing at least 50% of the lesion area; (3) Optical coherence tomography angiography (OCTA) demonstrating IRF and/or SRF; and (4) patients who received three consecutive monthly loading doses of faricimab at 6 mg (0.05 mL) after inadequate response to continuous anti-VEGF therapy. Exclusion criteria included: (1) significant ocular media opacity (e.g., cataract, vitreous hemorrhage) limiting visual or imaging assessment; (2) macular neovascularization secondary to non-AMD retinal disease; (3) other ocular disorders potentially affecting macular function (e.g., diabetic retinopathy, uveitis, epiretinal membrane); (4) evident vitreoretinal scarring or fibrosis; and (5) prior ocular interventions in the study eye other than anti-VEGF injections (e.g., macular laser, photodynamic therapy, intraocular surgery).

### Intervention and observation

All patients received a regimen of three monthly intravitreal injections (IVI) of faricimab (6 mg each). Comprehensive clinical evaluations were performed from baseline through 4 months after the third IVI. Best-corrected visual acuity (BCVA) was measured using a decimal chart, subsequently converted to logMAR units. Standardized ETDRS testing protocols were not consistently employed across all visits, which represents a methodological limitation of this real-world study. Fundus assessment was conducted via OCTA (RTVue XR Avanti, Optovue), utilizing standard depth protocols with acquisition of at least 80 vertical and horizontal B-scans centered on the fovea. All assessments were performed by the same trained technicians under consistent clinic lighting conditions to minimize variability.

### OCTA outcome assessment

OCTA images were acquired using the RTVue-XR Avanti OCT (Optovue, Inc., Fremont, CA, United States) with a 3 mm × 3 mm macular scan pattern. OCTA scans were inspected independently by two technicians, who manually refined retinal segmentation and verified correct assignment of vascular layers. Automated analysis produced macular thickness maps, partitioned using the ETDRS grid into central, inner, and outer subfields. Central subfield thickness (CST) was defined as the mean retinal thickness within the 1-mm central zone. The B-scan exhibiting maximal PED or RF was utilized; the region of interest was traced manually using the polygon tool, and area quantified via ImageJ. CNV area was ascertained on the outer-retinal to choriocapillaris slab utilizing an ImageJ macro following the methodology of Deshpande et al. (15).

### Statistical methods

All statistical analyses were performed using R (version 4.4.0). For independent group comparisons, normally distributed variables were analyzed by independent-samples *t*-test, while non-normally distributed variables employed the Wilcoxon rank-sum test. Paired data were assessed using paired *t*-tests or Wilcoxon signed-rank tests where appropriate; McNemar’s test was used for paired categorical variables. Correlations between baseline parameters and final BCVA were evaluated by Spearman’s correlation coefficient. Data are expressed as mean ± SD; statistical significance was defined as *p* < 0.05.

## Results

### Demographic and baseline characteristics

37 eyes from 37 patients with nAMD meeting inclusion criteria were enrolled. The mean age of the study population was 70.08 ± 8.60 years (range 57–84); 21 were male and the remainder female. Demographic and baseline characteristics are detailed in Table 1.

**Table 1:** Baseline demographics and clinical characteristics of enrolled patients with refractory nAMD.

Characteristic	Value
Sample size	37
Age, year	70.08 ± 8.60
Gender (M/F)	21/16
Laterality (R/L)	18/19
LogMAR BCVA	0.82 ± 0.42
CST (μm)	336.89 ± 141.52
No. of previous anti-VEGF injections	7.43 ± 4.71

M, male; F, female; R, right; L, left; BCVA, best-corrected visual acuity; CST, central subfield thickness.

### Effect of faricimab treatment

**Figure 1:**
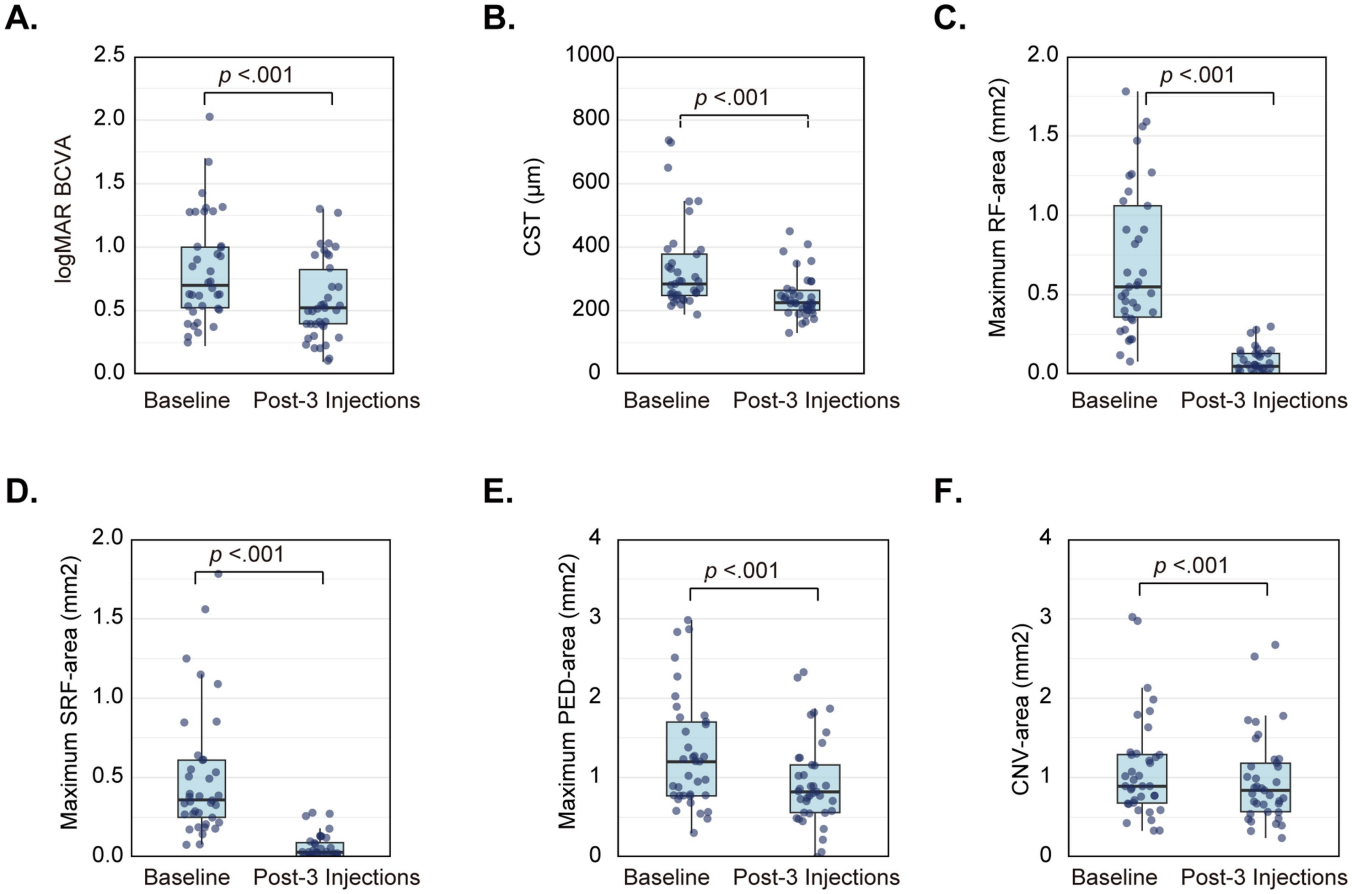
Changes in visual and anatomical outcomes in all 37 patients after three monthly faricimab injections. **(A)** LogMAR BCVA; **(B)** Central subfield thickness (CST); **(C)** Maximal retinal fluid (RF) area; **(D)** Maximal subretinal fluid (SRF) area; **(E)** Maximal pigment epithelial detachment (PED) area; **(F)** Choroidal neovascularization (CNV) area. Data are presented as mean ± SD. ***p < 0.001 (paired Wilcoxon signed-rank test).

Table 2 summarizes changes in visual acuity and OCTA parameters at baseline (Month 0) and after three monthly faricimab injections (Month 4). LogMAR BCVA decreased significantly from 0.82 ± 0.42 to 0.57 ± 0.32 (*p* < 0.001) (Figure 1A). Central subfield thickness decreased markedly from 336.8 ± 141.52 μm to 244.19 ± 69.91 μm (*p* < 0.001) (Figure 1B). Total RF area showed a substantial decline, with maximal RF area decreasing from 0.70 ± 0.46 to 0.08 ± 0.08 mm^2^ (*p* < 0.001) (Figure 1C). At baseline, all 37 eyes demonstrated SRF, but after treatment, SRF persisted in only 22 eyes (*p* = 0.004), and maximal SRF area was significantly reduced from 0.50 ± 0.40 to 0.06 ± 0.08 mm^2^ (*p* < 0.001) (Figure 1D). IRF was observed in 19 eyes at baseline and in 14 eyes after treatment, though this change was not statistically significant. PED area was reduced from 1.30 ± 0.71 to 0.94 ± 0.55 mm^2^ (*p* < 0.001) (Figure 1E). CNV area further decreased from 1.11 ± 0.64 to 0.97 ± 0.56 mm^2^ (*p* < 0.001) (Figure 1F). No statistically significant improvement was identified in the integrity of the ellipsoid zone (EZ) or external limiting membrane (ELM).

**Table 2:** Changes in BCVA and OCTA anatomical outcomes after three monthly doses of faricimab in patients with refractory nAMD.

	Baseline	Post-3 Injections	*p* value
LogMAR BCVA	0.82 ± 0.42	0.57 ± 0.32	<0.001*
CST (μm)	336.89 ± 141.52	244.19 ± 69.91	<0.001*
IRF (+/−)	19/18	14/23	0.074†
SRF (+/−)	37/0	22/15	<0.001†
Maximum SRF-area (mm^2^)	0.50 ± 0.40	0.06 ± 0.08	<0.001*
Maximum RF-area (mm^2^)	0.70 ± 0.46	0.08 ± 0.08	<0.001*
Maximum PED-area (mm^2^)	1.30 ± 0.71	0.94 ± 0.55	<0.001*
CNV-area (mm^2^)	1.11 ± 0.64	0.97 ± 0.56	<0.001*
Intact foveal EZ (+/−)	29/8	30/7	1.000†
Intact foveal ELM (+/−)	22/15	25/12	0.248†

BCVA, best-corrected visual acuity; CST, central subfield thickness; IRF, intraretinal fluid; SRF, subretinal fluid; RF, retinal fluid; PED, pigment epithelial detachment; CNV, choroidal neovascularization; EZ, Ellipsoid Zone; ELM, External Limiting Membrane. * Paired Wilcoxon rank-sum test, † McNemar’s test. *p* < 0.05 values indicate statistical significance.

Of the 37 eyes, 18 were categorized as having isolated SRF group, while the remaining 19 exhibited both SRF & IRF group. Baseline and post-treatment characteristics following three consecutive IVI faricimab injections are detailed in Table 3. The total RF area did not differ significantly between the groups at baseline (*p* = 0.704), but the SRF group had a larger baseline SRF area than the SRF & IRF group (*p* = 0.039). The mean number of prior anti-VEGF injections was similar (SRF: 7.28 ± 5.37; SRF & IRF: 7.58 ± 4.13), and there were no other significant baseline demographic differences.

**Table 3:** Baseline characteristics of refractory nAMD patients with SRF vs. SRF & IRF.

	SRF group	SRF & IRF group	*p* value
Sample size	18	19	N/A
Age, year	70.22 ± 9.47	69.95 ± 7.94	0.924†
Gender (M/F)	10/8	11/8	0.886*
Laterality (R/L)	7/11	11/8	0.248*
No. of previous anti-VEGF injections	7.28 ± 5.37	7.58 ± 4.13	0.562‡
LogMAR BCVA	0.74 ± 0.36	0.90 ± 0.46	0.278‡
CST (μm)	321.50 ± 97.18	351.47 ± 175.15	0.704‡
Maximum IRF-area (mm^2^)	0.00 ± 0.00	0.39 ± 0.30	<0.001‡
Maximum SRF-area (mm^2^)	0.66 ± 0.50	0.35 ± 0.20	0.039‡
Maximum RF-area (mm^2^)	0.66 ± 0.50	0.74 ± 0.42	0.224 ‡
Maximum PED-area (mm^2^)	1.32 ± 0.84	1.27 ± 0.58	0.952‡
CNV-area (mm^2^)	1.18 ± 0.70	1.04 ± 0.58	0.637‡

N/A, not applicable; M, male; F, female; R, right; L, left; BCVA, best-corrected visual acuity; CST, central subfield thickness; IRF, intraretinal fluid; SRF, subretinal fluid; RF, retinal fluid; PED, pigment epithelial detachment; CNV, choroidal neovascularization. * Chi-square test, † Two-sample *t*-test, ‡ Wilcoxon rank-sum test. *p* < 0.05 values indicate statistical significance.

**Figure 2:**
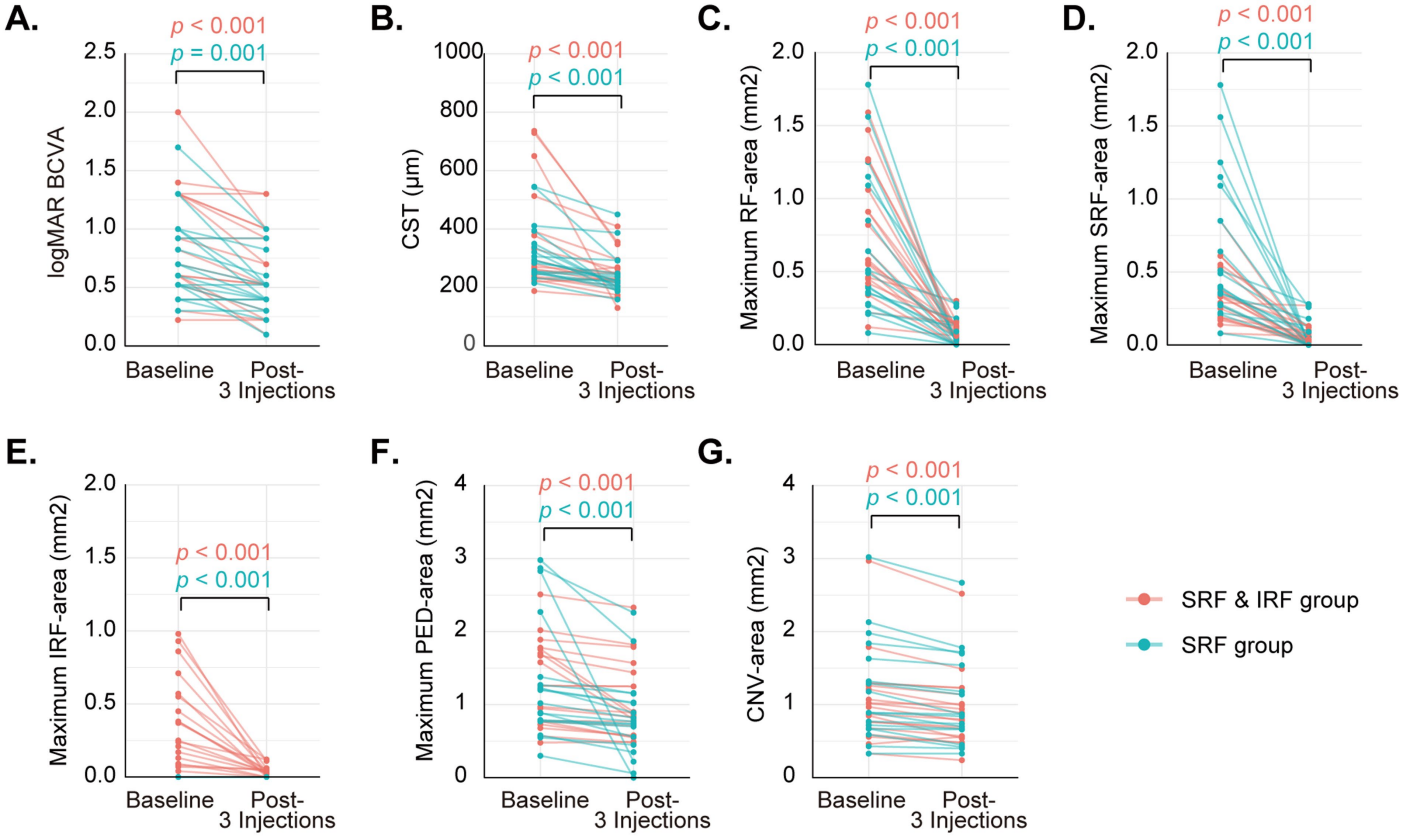
Comparison of outcomes between the isolated SRF group (*n*=18) and the SRF & IRF group (*n*=19) after three monthly faricimab injections. **(A)** LogMAR BCVA; **(B)** CST; **(C)** Maximal RF area; **(D)** Maximal SRF area; **(E)** Maximal IRF area; **(F)** Maximal PED area; **(G)** CNV area. *p < 0.05, ***p < 0.001 (paired Wilcoxon signed-rank test for within-group changes; between-group comparisons were non-significant for all parameters as shown in Table 4).

After three monthly faricimab injections, changes in BCVA, CST, total RF, SRF, PED, and CNV areas did not differ significantly between the two groups (Table 4). Nevertheless, both subgroups experienced significant improvement in BCVA (*p* = 0.001 for SRF group*, p* < 0.001 for SRF & IRF group; Figure 2A). In the SRF group, CST (*p* < 0.001; Figure 2B), maximal RF area (*p* < 0.001; Figure 2C), maximal SRF area (*p* < 0.001; Figure 2D), maximal PED area (*p* < 0.001; Figure 2F) and CNV area (*p* < 0.001; Figure 2G) all showed marked decreases. The SRF & IRF group similarly exhibited significant reductions in CST (*p* < 0.001; Figure 2B), maximal RF area (*p* < 0.001; Figure 2C), maximal SRF area (*p* < 0.001; Figure 2D), maximal IRF area (*p* = 0.018; Figure 2E), maximal PED area (*p* < 0.001; Figure 2F) and CNV area (*p* < 0.001; Figure 2G).

**Table 4:** Four-month outcomes following three faricimab loading doses in refractory nAMD patients: comparison between SRF and SRF & IRF subgroups.

Parameter	SRF group	SRF & IRF group	*p* value
LogMAR BCVA	0.48 ± 0.23	0.65 ± 0.37	0.187*
CST (μm)	242.72 ± 73.26	245.58 ± 68.56	0.504*
Maximum IRF-area (mm^2^)	0.00 ± 0.00	0.04 ± 0.04	<0.001*
Maximum SRF-area (mm^2^)	0.07 ± 0.09	0.06 ± 0.11	0.787*
Maximum RF-area (mm^2^)	0.07 ± 0.09	0.09 ± 0.07	0.097*
Maximum PED-area (mm^2^)	0.82 ± 0.57	1.04 ± 0.53	0.213*
CNV-area (mm^2^)	1.04 ± 0.62	0.91 ± 0.50	0.738*

BCVA, best-corrected visual acuity; CST, central subfield thickness; IRF, intraretinal fluid; SRF, subretinal fluid; RF, retinal fluid; PED, pigment epithelial detachment; CNV, choroidal neovascularization. * Wilcoxon rank-sum test. *p* < 0.05 values indicate statistical significance.

**Figure 3:**
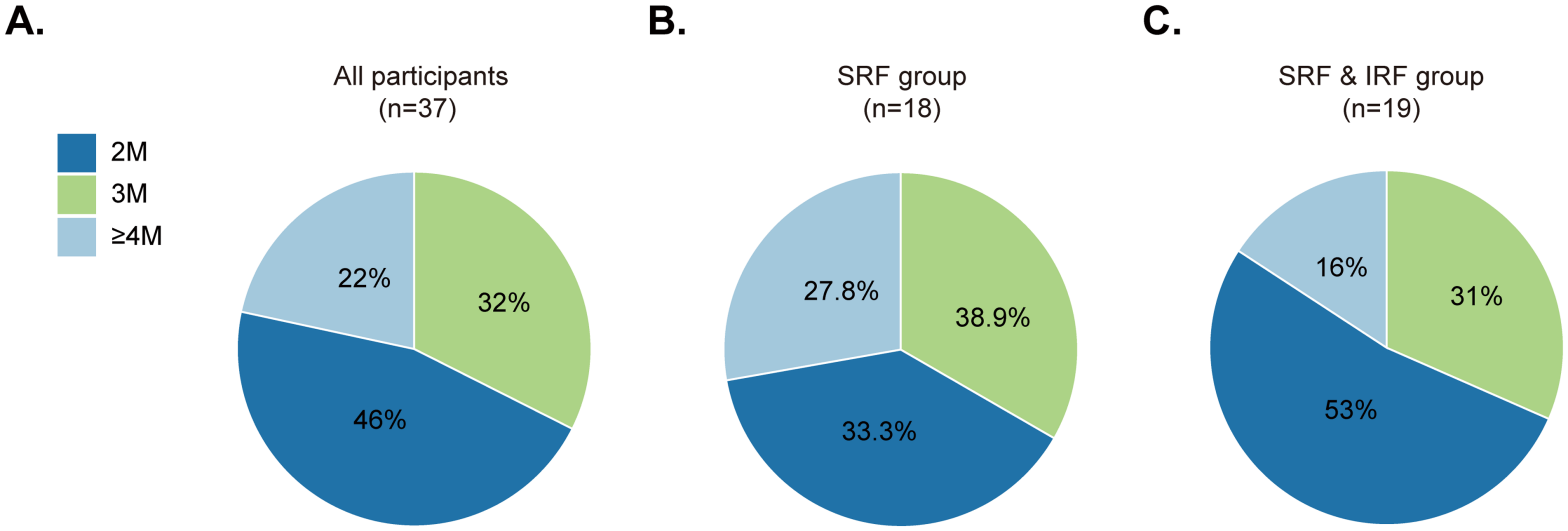
Distribution of minimum retreatment intervals after three monthly loading doses of faricimab. Proportional pie charts show the percentage of eyes achieving dosing intervals of 2, 3, or 4 months. **(A)** All patients (*n*=37); **(B)** Isolated SRF group (*n*=18); **(C)** SRF & IRF group (*n*=19).

After three monthly faricimab injections, all eyes achieved a minimum retreatment interval exceeding 2 months. 54% of eyes were able to extend their retreatment interval to ≥3 months, with 22% reaching an interval of ≥4 months (Figure 3A). In the SRF group, 66.7% attained a dosing interval of ≥3 months and 27.8% reached ≥4 months (Figure 3B). By contrast, approximately 47% of eyes in the SRF & IRF group extended to ≥3 months and about 16% achieved ≥4 months (Figure 3C). These results suggest that faricimab may enable longer dosing intervals, particularly in cases with isolated SRF.

### Safety

Faricimab exhibited an excellent safety profile in this cohort. No serious adverse events—such as endophthalmitis, retinal detachment, pronounced intraocular pressure elevation, or vitreous hemorrhage—were observed. Importantly, no cases of drug-related retinal vasculitis, retinal arterial occlusion, or retinal pigment epithelium (RPE) tear occurred. Only mild, transient, injection-related adverse events (e.g., conjunctival hemorrhage/hyperemia, ocular discomfort) were noted and resolved spontaneously. Collectively, these data support the favorable tolerability and safety of faricimab for refractory nAMD.

## Discussion

As an exploratory, real-world observational study without a comparator arm, the following findings should be interpreted as hypothesis-generating rather than conclusive. To our knowledge, this is one of the few real-world studies specifically evaluating faricimab in Chinese patients with refractory nAMD. This study reports on the short-term outcomes of intravitreal faricimab therapy in Chinese patients with refractory nAMD, highlighting valuable real-world evidence regarding its efficacy and safety. The findings demonstrate that faricimab confers significant short-term efficacy, including pronounced reductions in mean CST, marked improvement in BCVA, increased proportions of patients becoming free of SRF and RF, and prolonged treatment intervals after the three-dose loading phase. The mechanistic advantage of faricimab—targeting both VEGF-A and Ang-2 pathways—likely underlies its substantial effectiveness in refractory nAMD. While switching to alternative anti-VEGF agents such as aflibercept or brolucizumab has been reported to yield modest visual or anatomical gains in some studies, the current single-arm study design does not allow direct comparisons with other anti-VEGF agents. Whether the dual inhibition provided by faricimab offers additional benefits in vascular stabilization, recurrence reduction, or disease control compared to existing therapies remains to be determined in future head-to-head comparative trials. It is important to recognize that refractory nAMD patients differ fundamentally from treatment-naïve populations, as chronic fluid persistence often leads to irreversible structural damage that limits the potential for substantial visual recovery.

Among patients with refractory nAMD in this cohort, switching to faricimab resulted in significant improvement in BCVA (from 0.82 ± 0.42 to 0.57 ± 0.32 logMAR, *p* < 0.001) and CST (from 336.89 ± 141.52 μm to 244.19 ± 69.91 μm, *p* < 0.001). These findings are qualitatively consistent with results from global clinical trials which demonstrated non-inferior BCVA gains and CST reductions with faricimab compared to aflibercept in treatment-naïve nAMD patients (16). However, direct numerical comparisons between our study and those trials are not warranted, given the substantial differences in patient populations and the uncontrolled, real-world nature of our study. In a real-world prospective observational study of 37 refractory nAMD eyes, three monthly IVI of faricimab led to an improvement in BCVA from 0.84 ± 0.49 to 0.76 ± 0.49 logMAR, with a 27% decrease in central macular thickness (from 447.65 ± 161.46 μm to 327.33 ± 147.78 μm) (17). Notably, BCVA improvements were observed across the entire cohort, underscoring faricimab’s potential as an effective therapy for various nAMD manifestations. Although atypical fluid responses following anti-VEGF monotherapy have been documented in some nAMD phenotypes, including persistent or paradoxical fluid accumulation, the distribution of nAMD subtypes did not meaningfully influence the extent of vision improvement after IVI in this study (18, 19). Baseline BCVA and patient age have been reported as predictors of visual outcomes in other nAMD studies (17), though the sample size of the present study precluded robust multivariable analysis. Other studies have reported that central retinal thickness, maximum subretinal hyperreflective material height, and foveal external limiting membrane (ELM), IRF, and SRF status are also predictive factors for final visual outcomes (20, 21).

PED is observed in between 30 and 80% of nAMD cases (22). It is typically caused by accumulation of SRF and/or IRF as well as macular neovascularization (MNV), which leads to displacement of the RPE from Bruch’s membrane, producing a range of clinical presentations. In the present study, maximal PED thickness was significantly reduced in patients with refractory nAMD treated with faricimab (*p* < 0.001), consistent with findings of Liu et al. (23). Literature suggests that baseline PED height, extent of vascularization, and the choice of anti-VEGF agent all impact the PED response (24). Importantly, rapid PED resolution has been linked to superior long-term visual outcomes, thus emphasizing the importance of effective management (25). Both VEGF and Ang-2 are central contributors to MNV in nAMD, with Ang-2 also regulating vascular permeability (26–29). The present study demonstrated a significant decrease in CNV area after faricimab administration (*p* < 0.001). This supports the hypothesis that dual inhibition of VEGF-A and Ang-2 by faricimab may surmount resistance mechanisms found with anti-VEGF monotherapy.

SRF and IRF are recognized as robust biomarkers of macular neovascularization activity in both treatment-naïve and previously treated patients (30–33). Persistent or recurrent fluid accumulation during anti-VEGF therapy is directly linked to progressive vision loss over time (34–38). The resolution of RF, particularly in the early treatment course, is an objective marker of disease control or inactivity. Results from global phase 3 clinical trials indicate that dual-pathway inhibition by faricimab led to nearly 80% of patients achieving fluid resolution following the loading phase. Moreover, the drug conferred sustained durability during maintenance, with 80% of patients extending retreatment intervals to ≥3 months, 60% to ≥4 months, and 50% to 5 months or more (16). Our study similarly demonstrated marked decreases in both RF and SRF after faricimab. This is an important result, as chronic macular fluid is a hallmark of refractory nAMD and highly predictive of long-term visual deterioration. Ang-2 inhibition may further promote vascular stabilization and attenuate inflammation, potentially addressing mechanisms not fully controlled by VEGF blockade alone. Our findings concur with those of Tanaka et al. and others. Of particular note, fluid reduction may translate to sustained vision preservation and enhanced quality of life for AMD patients. The TENAYA/LUCERNE trials also documented faster achievement of dry macula with faricimab compared to aflibercept in treatment-naïve populations (39). However, whether similar speed of fluid resolution occurs in refractory patients requires further investigation. In the present study, following the loading phase, all patients maintained a treatment-free interval of at least 2 months. Among those requiring retreatment, 46% were retreated at 2 months, 32% at 3 months, and 22% at 4 months or beyond. Subgroup analysis revealed that in the SRF-only cohort, 66.7% attained a dosing interval of ≥3 months and 27.8% reached ≥4 months. Meanwhile, in the SRF & IRF group, approximately 47% of eyes extended their interval to ≥3 months and 16% achieved ≥4 months. To our knowledge, this is the first report on retreatment intervals post-loading in Chinese refractory nAMD patients. These data reinforce the benefit of a treat-and-extend (T&E) individualized approach, emphasizing that retreatment intervals should be adjusted based on ongoing anatomical assessment with OCT at each visit, rather than using a fixed schedule. The reduction in IRF presence was not statistically significant, suggesting a more limited effect of faricimab on IRF compared with SRF. Given that IRF is a biomarker of poorer prognosis than isolated SRF, this modest response warrants cautious interpretation, and outcomes in patients with baseline IRF should be carefully considered.

The safety profile of faricimab has been further characterized by a real-world pharmacovigilance study using the FAERS database (40, 41). The safety profile of faricimab in our cohort aligned with that established in global studies and with other anti-VEGF therapies. In this small cohort, no serious adverse events such as endophthalmitis, retinal detachment, or vitreous hemorrhage were observed during the four-month follow-up period. However, the sample size and follow-up duration are insufficient to rule out rare adverse events.

The primary limitations of this study are the small sample size and the short follow-up duration. A key limitation is that switching to faricimab and shortening the injection interval to 4 weeks occurred simultaneously; therefore, we cannot determine whether improvements were due to the drug switch or the increased injection frequency. A control group continuing prior anti-VEGF at 4-week intervals would be needed to answer this question. Consequently, the observed anatomical improvements, while positive, are largely expected given the intensive monthly loading regimen administered to all patients, regardless of the specific anti-VEGF agent used. The current follow-up period is insufficient to draw definitive conclusions regarding long-term durability, disease recurrence control, or sustained feasibility of extending treatment intervals under a real-world treat-and-extend paradigm. The four-month follow-up precludes assessment of potential late responders, so the current findings may underestimate faricimab’s cumulative benefit in some patients, warranting longer-term studies. Our findings should thus be interpreted as short-term, exploratory results that require validation in larger, prospective cohorts with extended follow-up. Additionally, as a single-arm observational study without a comparator group, our findings cannot directly establish the superiority of faricimab over other anti-VEGF agents, and any comparative interpretations should be avoided.

In summary, our short-term study suggests that intravitreal faricimab is an effective and safe therapeutic alternative for Chinese patients with refractory nAMD. Its dual mechanism of action appears to confer substantial anatomical and functional benefit in eyes unresponsive to prior anti-VEGF agents. Moreover, faricimab’s ability to facilitate longer retreatment intervals may help alleviate overall patient treatment burden. Faricimab represents a significant innovation in our therapeutic armamentarium, supporting the advancement toward more personalized, effective nAMD management.

## Data Availability

The raw data supporting the conclusions of this article will be made available by the authors, without undue reservation.
